# Spatiotemporal Analysis of the Prevalence and Pattern of Multimorbidity in Older Chinese Adults

**DOI:** 10.3389/fmed.2021.806616

**Published:** 2022-01-20

**Authors:** Shimin Chen, Shengshu Wang, Wangping Jia, Ke Han, Yang Song, Shaohua Liu, Xuehang Li, Miao Liu, Yao He

**Affiliations:** ^1^Institute of Geriatrics, Beijing Key Laboratory of Aging and Geriatrics, National Clinical Research Center for Geriatric Disease, Chinese People's Liberation Army Medical School, Second Medical Center of Chinese People's Liberation Army General Hospital, Beijing, China; ^2^School of Non-commissioned Officer, Army Medical University, Hebei, China; ^3^Department of Gastroenterology, Chinese People's Liberation Army Medical School, First Medical Center of Chinese People's Liberation Army General Hospital, Beijing, China; ^4^Department of Statistics and Epidemiology, Graduate School, Chinese People's Liberation Army Medical School, Chinese People's Liberation Army General Hospital, Beijing, China

**Keywords:** multimorbidity, pattern, prevalence, older adults, LMICs

## Abstract

**Background:**

Multimorbidity presents an enormous problem to societal and healthcare utilization under the context of aging population in low- and middle-income countries (LMICs). Currently, systematic studies on the profile of multimorbidity and its characteristics among Chinese elderly are lacking. We described the temporal and spatial trends in the prevalence of multimorbidity and explored chronological changes of comorbidity patterns in a large elderly population survey.

**Methods:**

Data were extracted from the Chinese Longitudinal Healthy Longevity Study (CLHLS) conducted between 1998 and 2018 in a random selection of half of the counties and city districts. All the elderly aged 65 and older were included in the survey of eight waves. We used 13 investigated chronic diseases to measure the prevalence of multimorbidity by means of geography, subpopulation, and chronological changes. The patterns of multimorbidity were assessed by computing the value of relative risk (RR indicates the likelihood of certain diseases to be associated with multimorbidity) and the observed-to-expected ratio (O/E indicates the likelihood of the coexistence of a multimorbidity combination).

**Results:**

From 1998 to 2018, the prevalence of multimorbidity went from 15.60 to 30.76%, increasing in the fluctuation across the survey of eight waves (*p*_for trend_ = 0.020). Increasing trends were observed similarly in a different gender group (*p*_male_ = 0.009; *p*_female_ = 0.004) and age groups among female participants (*p*_~80_ = 0.009; *p*_81−90_ = 0.004; *p*_91−100_ = 0.035; *p*_101~_ = 0.018). The gap in the prevalence of multimorbidity between the north and the south was getting narrow across the survey of eight waves. Hypertension was the highest prevalent chronic condition while diabetes was most likely to coexist with other chronic conditions in the CLHLS survey. The most frequently occurring clusters were hypertension and heart disease, hypertension and cataract, and hypertension and chronic lung disease. And, the cancer, TB, and Parkinson's disease cluster took the domination of O/E rankings over time, which had a higher probability of coexistence in all the multimorbidity combinations.

**Conclusions:**

The prevalence of multimorbidity has been increasing nationwide, and more attention should be paid to a rapid growth in the southern part of China. It demands the effective diagnosis and treatment adopted to the highly prevalent comorbidities, and strategies and measures were adjusted to strongly relevant clusters.

## Highlights

- Our findings indicated an increasing trend with fluctuations in the prevalence of multimorbidity among the older Chinese adults, as is similar to different age, sex, and region groups.
- We identified several important disease combinations, including a highly prevalent hypertension cluster, likely to coexist the diabetes cluster and the cancer, TB, and Parkinson's disease cluster.
- Our study indicated further etiological studies, the generation of prevention strategies, and the formulation of public health policies on the multimorbidity for LMICs.

## Introduction

Multimorbidity is defined as the coexistence of more than two chronic diseases or long-term conditions, which can only be controlled by medications or other treatments and should not be treated in isolation (1). Multimorbidity has emerged as a substantial challenge to the global health system presently and in the coming decades, in association with high mortality (2), reducing quality of life (3), resulting in frailty (4), and other major consequences (5, 6). A few studies showed that there were more people with multimorbidity than those with single disease alone in a large-scale and national cross-sectional study (7). And, the complexities in multimorbidity make it increasingly difficult to provide optimal care, existing health systems are dominated by single-disease approaches in which patients with multimorbidity are treated in a duplicative and an isolated approach (8). Therefore, the importance of studies on multimorbidity should be emphasized, facilitating the implementation of comprehensive treatment.

The issue of multimorbidity is partly driven by the aging of the global population, including having social care implications on account of the risk of function decline and loss of independence with older age (9). Aging is usually accompanied by chronic disease (10), and it developed multimorbidity (11, 12) eventually. China, as the largest middle-income country, went through a rapid economic development as the reform and opening up in the past 20 years. Similar to most low- and middle-income countries (LMICs) around the world, China has witnessed an unprecedented upward swift in life expectation and the stage of aging population (13). The number of older people aged between 65+ and 80+ in China is expected to grow to 400 and 150 million by 2050 (14), respectively. The aging demographic transformation resulting in a high prevalence of multiple chronic diseases or conditions presents an enormous problem to societal and healthcare utilization (15).

Previous evidence on multimorbidity was based on the cross-sectional studies of sampling specific populations in various settings, in which studies covering representative nationwide sample size are far from adequate (16, 17). There is no evidence on the variation trends and pattern analysis based on a longitudinal study design in LMICs, which is conducive to global public health actions. Our purpose was to describe the temporal and spatial trends in the prevalence of multimorbidity and explore chronological changes of comorbidity patterns in a large elderly population survey.

## Materials and Methods

### Sample and Data

The data of this study were derived from the eight waves of the Chinese Longitudinal Healthy Longevity Study (CLHLS), which is the earliest and longest social science survey in China. Briefly, the CLHLS collected longitudinal data on the elderly aged 65, and it was over-coordinated by the Center for Healthy Aging and Development Studies of National School of Development at Peking University. The surveys were started from 1998 and followed up in 2000, 2002, 2005, 2008, 2011, 2014, and 2018, covering about half of the counties and city districts in 23 of the 34 Chinese provinces. More details about CLHLS were described elsewhere (18). The data include individual weighting variables to ensure whether they are nationally representative. For this study, there were 9,093, 11,199, 16,064, 15,638, 16,954, 9,765, and 7,192 participants included in each wave survey after processing the missing values. The demographic characteristics of sample respondents across the eight waves are presented in Supplementary Table 1.

### Variables

The questionnaire data provided information on demographic factors, physiological and psychological health status, the prevalence of chronic disease, and socioeconomic characteristics. Face-to-face home-based interviews were conducted by the trained staff. For this study, all participants who aged more than 65 years were included with complete records on essential information. The essential information consisted of age, gender, residence, province, and chronic diseases or conditions.

As shown in Supplementary Table 2, the chronic diseases or conditions investigated across the eight surveys included the following 13 self-reported chronic diseases or conditions investigated in each wave: hypertension, diabetes, heart disease, stroke or CVD, chronic lung disease (bronchitis, emphysema, pneumonia, and asthma), TB, cataract, glaucoma, cancer, prostate tumor, gastric or duodenal ulcer, Parkinson's disease, and bedsore. Accordingly, acute or subacute forms of certain conditions were excluded by the CLHLS protocol. Multimorbidity was calculated by using the criterion of ≥2 conditions of the 13 chronic conditions list (7). The prevalence of single disease and multimorbidity was weighted according to the sampling probabilities, which were based on the age–sex–residence-specific distribution from the CLHLS study.

### Statistical Analysis

We described the demographic characteristics across the eight CLHLS survey waves to understand the whole situation of the study population. Descriptive statistics were analyzed and presented as medians [interquartile range (IQR)] for non-normally distributed continuous data and frequencies (percentage) for categorical data.

We calculated the prevalence of multimorbidity by the totality and subpopulations across the survey of eight waves. Weight was calculated based on the age–sex–residence-specific distribution from the CLHLS study. In addition to weight total prevalence, χ^2^ test, and Mann–Kendall trend test were applied to identify the difference of multimorbidity in the subpopulation and chronological change, respectively. To visualize the trends in the prevalence of multimorbidity, percentage bar plot, pyramid of multimorbidity, and the map of distribution were drawn by the packages “ggplot2” and “plotrix.”

To explore the patterns of multimorbidity, the first step was to find out the trends in the prevalence of singular conditions. We computed the weighted prevalence of singular conditions using the waves. The second step was to calculate Relative risk (RR), which was calculated as the prevalence of certain diseases in the multimorbidity group divided by the prevalence of certain diseases in the non-multimorbidity group. The higher the RR value, the higher the probability of the disease coexisting with other diseases. The heatmap of RR of singular conditions and of a biaxial figure was presented by the packages “plotrix” and “pheatmap,” respectively.

The final step was to compute the observed-to-expected (O/E) ratio defined as an indicator for assessing the correlation between diseases or conditions, which is determined as Observed prevalence divided by Expected prevalence. Expected prevalence of multimorbidity combination equals the product of the prevalence of separate diseases if these diseases are completely independent of each other. The probability of coexistence of chronic conditions increased with the elevation of the O/E value. We calculated 78 dyad combinations possible given the 13 different chronic conditions considered in this study and presented the five highest O/E multimorbidity combinations.

All the analyses were performed using Excel (version 2019) and R (version 4.0.3). The values of *p* < 0.05 were considered to be statistically significant.

## Result

### Trends in Prevalence of Multimorbidity

**Figure 1:**
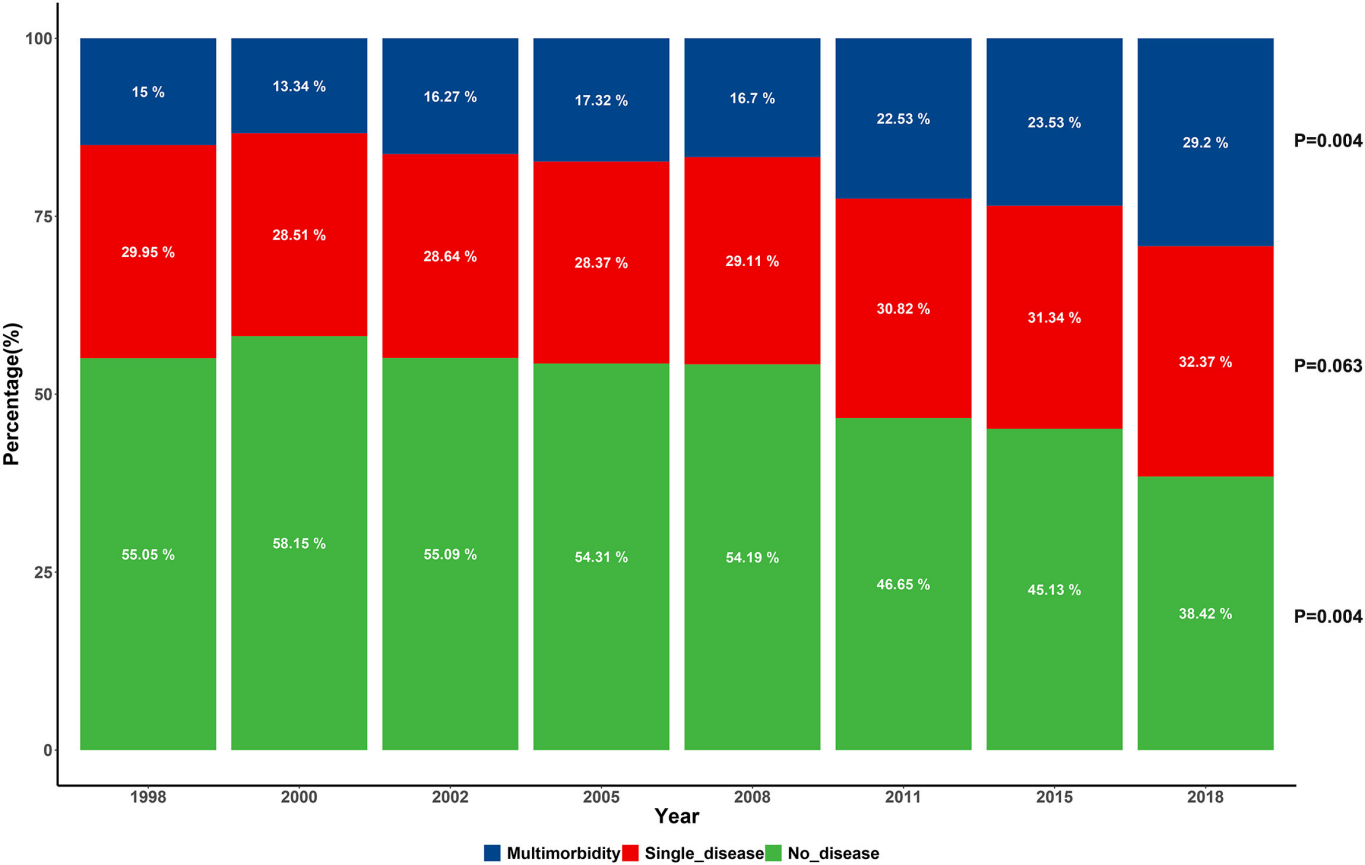
Percentage of the elderly suffering from the various number of chronic conditions across the survey of eight waves.

**Figure 2:**
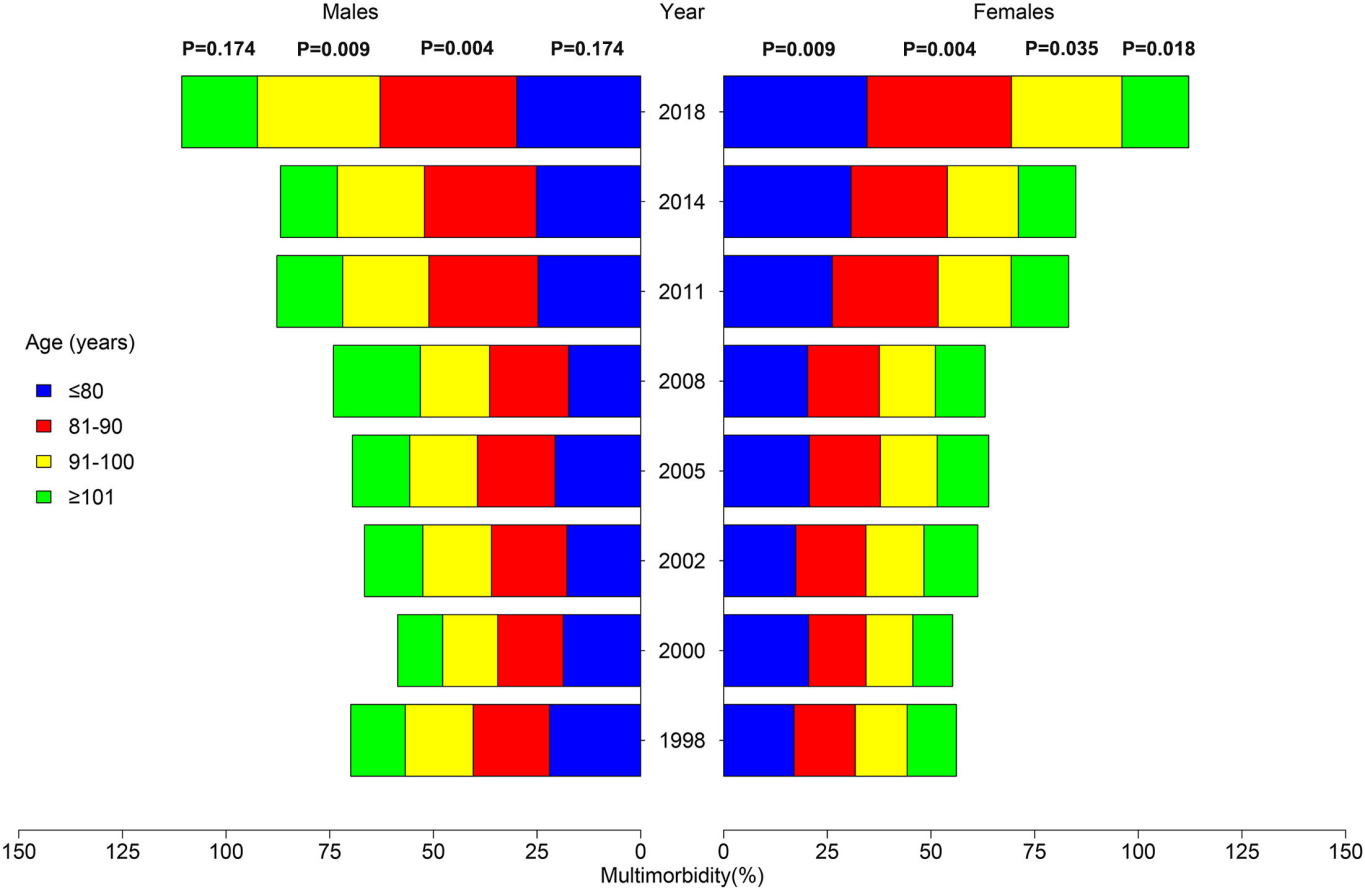
Pyramid of multimorbidity by gender and age catalog.

There is an increasing tendency with the fluctuation in the adjusted prevalence of multimorbidity, which increased from 15.60% in 1998 to 30.76% in 2018 (*p* = 0.020). Figure 1 shows the proportion of participants aged over 65 according to morbidity numbers. The proportion of respondents who suffered from none of the 13 chronic conditions decreased from 55.05% in 1998 to 38.42% in 2018 (*p* = 0.004). The prevalence of multimorbidity in the subpopulation is displayed in Table 1. Similar to the total population, there was also a significant increasing trend in both male (*p* = 0.009) and female (*p* = 0.004). The prevalence of multimorbidity was relatively high in the 65–80-year-old group across time. There was a significant rise in tendency in the 81–90- (*p* = 0.004) and 91–100-year-old groups (*p* = 0.025). Further subpopulation analysis of multimorbidity pyramid is illustrated in Figure 2 and Supplementary Table 3. It can be seen that the trend was observed to be significant in each age group among female participants (*p*_~80_ = 0.009; *p*_81−90_ = 0.004; *p*_91−100_ = 0.035; *p*_101~_ = 0.018).

**Table 1:** Prevalence of multimorbidity by the subpopulations across the survey of eight waves.

**Wave**	**1998**	**2000**	**2002**	**2005**	**2008**	**2011**	**2014**	**2018**	^*^P_**for trend**_
Total	1,364 (15.00)	1,494 (13.34)	2,614 (16.27)	2,708 (17.32)	2,831 (16.70)	2,200 (22.53)	1,692 (23.53)	4,636 (29.20)	0.004
**Gender**									
Male	638 (17.53)	689 (14.81)	1,190 (17.38)	1,247 (18.65)	1,304 (17.98)	1,051 (23.90)	813 (24.52)	2,072 (29.92)	0.009
Female	726 (13.31)	805 (12.29)	1,424 (15.45)	1,461 (16.32)	1,572 (15.74)	1,149 (21.41)	879 (22.68)	2,564 (28.65)	0.004
*P*	<0.001	<0.001	<0.001	<0.001	<0.001	0.003	0.067	0.081	
**Age**									
~80	110 (19.54)	121 (19.48)	916 (17.59)	1,091 (20.66)	961 (18.73)	880 (25.41)	746 (27.67)	1,908 (32.24)	0.063
81–90	576 (16.59)	732 (14.81)	768 (17.57)	758 (17.92)	827 (18.16)	689 (25.95)	548 (25.09)	1,403 (33.95)	0.004
91–100	496 (13.95)	469 (12.05)	627 (14.95)	595 (14.69)	695 (14.69)	455 (18.88)	302 (18.67)	990 (26.03)	0.025
101~	182 (12.10)	172 (9.87)	303 (13.23)	2,078 (12.70)	2,537 (13.72)	176 (14.23)	96 (13.83)	335 (16.60)	0.174
*P*	<0.001	<0.001	<0.001	<0.001	<0.001	<0.001	<0.001	<0.001	
**Residence**									
City	691 (19.92)	748 (22.08)	1,034 (26.89)	1,117 (28.80)	1,075 (32.08)	663 (38.21)	403 (40.79)	1,727 (48.76)	0.003
Town	-	348 (9.91)	550 (15.50)	444 (14.32)	450 (13.60)	638 (22.11)	541 (24.33)	1,308 (24.97)	0.764
Rural	673 (11.97)	398 (9.26)	1,030 (11.88)	1,147 (13.25)	1,306 (12.69)	899 (17.47)	748 (18.79)	1,601 (22.57)	0.007
*P*	<0.001	<0.001	<0.001	<0.001	<0.001	<0.001	<0.001	<0.001	
^**^Weighted total	15.60 (14.85–16.38)	13.43 (12.80–14.09)	16.13 (15.56–16.72)	19.93 (19.32–20.56)	19.93 (19.32–20.55)	24.39 (23.53–25.28)	23.36 (22.37–24.39)	30.76 (30.03–31.49)	0.020

*-The survey conducted in 1998 did not subdivide the urban group into cities and towns*.

* *Mann–Kendall trend test*.

** *Weight was calculated based on the age–sex–residence-specific distribution from the Chinese Longitudinal Healthy Longevity Study (CLHLS) study, presented as the rate (95% CI)*.

**Figure 3:**
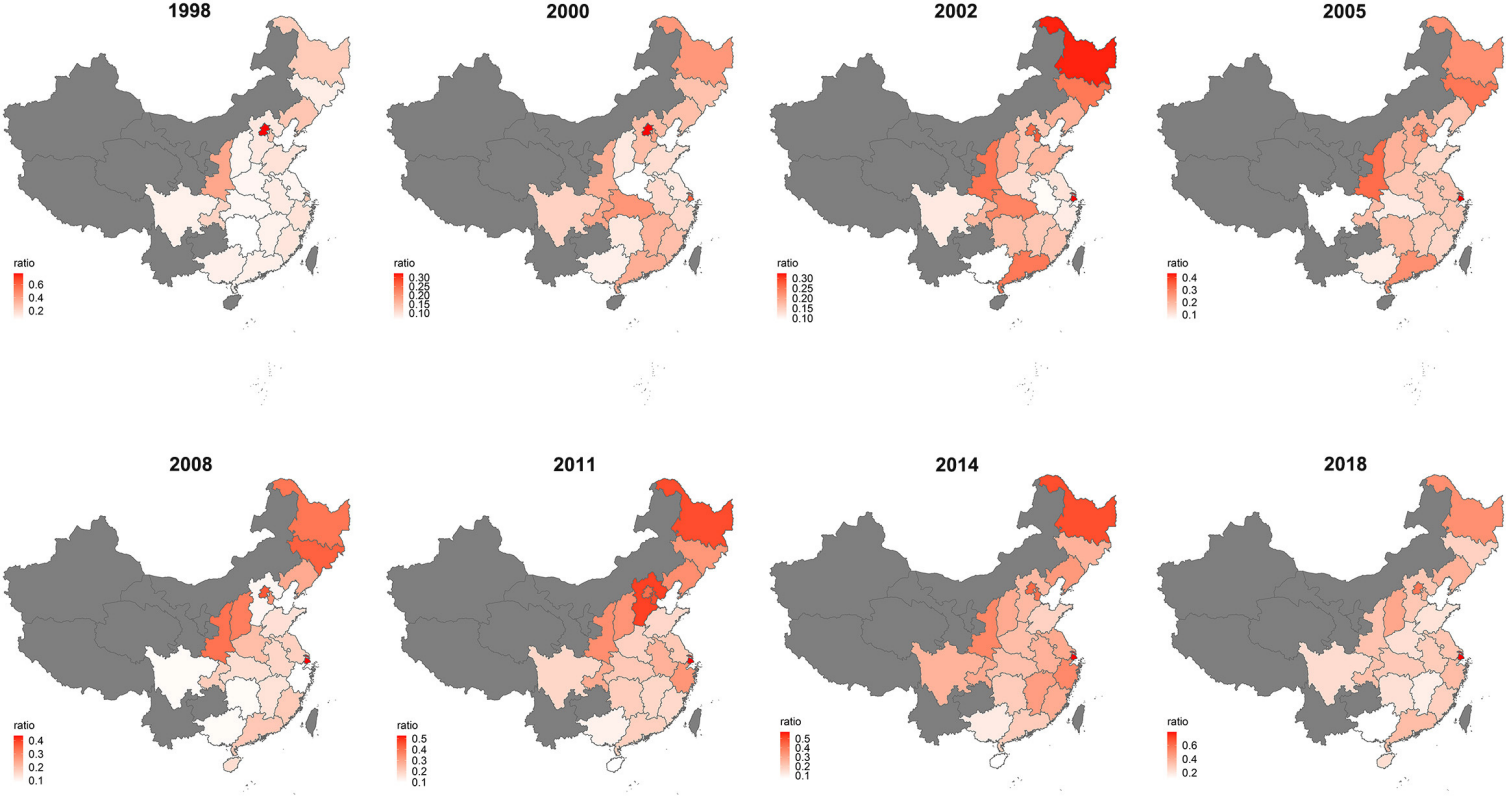
The distribution of prevalence in different provinces for the survey of eight waves.

The spatial distribution of multimorbidity is shown in Figure 3. There was an indication that Beijing has the highest prevalence of multimorbidity in the first two survey waves (76.99%; 32.3%), and Shanghai leads in the following six survey waves. Its prevalence in the northeastern and north central part was higher than the southern part in China during 1998–2008, and the tendency became average till 2018. Supplementary Table 4 shows the prevalence of multimorbidity with the surveyed province across the eight waves. In the 1998 wave, 26.68% of the participants had multimorbidity in Shanghai and the proportion rose to 79.50% in the 2018 wave, an increasing trend was significant (*p* = 0.002). Similar trends were observed in Heilongjiang (*p* = 0.004), Shanxi (*p* = 0.009), Jiangsu (*p* = 0.004), Anhui (*p* = 0.019), Fujian (*p* = 0.035), Shandong (*p* = 0.009), and Hunan (*p* = 0.009).

### Trends in Prevalence of Chronic Conditions

Table 2 shows the weighted prevalence of single chronic disease or condition among the eight waves. Hypertension being the highest prevalence of all chronic conditions increased from 17.27% in 1998 to 44.33% in 2018. A similar increasing trend along with the eight waves in diabetes, heart disease, stroke or CVD, cancer, prostate tumor, and gastric or duodenal ulcer was observed. The decreasing trend was shown in the prevalence of the rest of conditions. For example, the prevalence of chronic lung disease decreased with the fluctuation from 13.73% in 1998 to 8.52% in 2018. As for Parkinson's disease, the highest prevalence was 1.13% in 2011 while the lowest was 0.3% in 2002 in the process of chronological change.

**Table 2:** Weighted prevalence of singular chronic disease or condition by the survey of eight waves.

**Prevalence (%)**	**1998**	**2000**	**2002**	**2005**	**2008**	**2011**	**2014**	**2018**
Hypertension	17.27 (16.49–18.08)	17.70 (16.99–18.43)	17.72 (17.13–18.32)	22.94 (22.29–23.60)	25.26 (24.60–25.94)	33.68 (28.18)	33.46 (32.34–34.59)	44.33 (43.55–45.12)
Diabetes	1.81 (0.97–1.44)	1.64 (1.42–1.90)	3.23 (2.96–3.52)	4.20 (3.89–4.52)	4.74 (4.42–5.08)	7.06 (6.55–7.60)	7.88 (7.26–8.54)	12.54 (12.03–13.08)
Heart disease	7.85 (7.30–8.43)	7.72 (7.23–8.24)	10.23 (9.76–10.71)	11.27 (10.78–11.77)	12.20 (11.70–12.71)	13.64 (12.95–14.35)	13.38 (12.59–14.21)	15.96 (15.38–16.54)
Stroke or CVD	3.54 (3.16–3.95)	3.87 (3.52–4.25)	5.97 (5.60–6.35)	6.49 (6.12–6.88)	7.38 (6.98–7.79)	7.87 (7.33–8.43)	8.85 (8.19–9.55)	10.65 (10.18–11.15)
Chronic lung disease	13.73 (13.02–14.47)	11.82 (11.22–12.44)	12.66 (12.15–13.19)	12.23 (11.73–12.75)	10.20 (9.74–10.68)	10.50 (9.89–11.14)	9.18 (8.51–9.90)	8.52 (8.09–8.97)
Tuberculosis	0.87 (0.69–1.09)	0.94 (0.77–1.15)	0.90 (0.77–1.07)	0.95 (0.81–1.12)	0.77 (0.65–0.92)	1.09 (0.89–1.32)	0.47 (0.32–0.67)	0.57 (0.46–0.70)
Cataract	15.31 (14.56–16.08)	9.56 (9.02–10.13)	7.24 (6.85–7.66)	8.03 (7.62–8.46)	8.25 (7.83–8.69)	8.22 (7.68–8.80)	8.35 (7.71–9.04)	9.66 (9.20–10.14)
Glaucoma	2.03 (1.75–2.35)	2.03 (1.77–2.31)	1.75 (1.55–1.97)	1.74 (1.54–1.95)	1.89 (1.69–2.11)	1.48 (1.25–1.75)	1.20 (0.96–1.49)	1.53 (1.34–1.73)
Cancer	0.55 (0.31–0.72)	0.18 (0.11–0.29)	0.55 (0.44–0.68)	0.68 (0.56–0.82)	0.58 (0.47–0.72)	0.85 (0.68–1.06)	0.79 (0.60–1.04)	1.46 (1.28–1.66)
Prostate tumor	3.47 (3.10–3.88)	1.77 (1.54–2.04)	1.78 (1.58–2.00)	3.04 (2.78–3.32)	3.65 (3.37–3.95)	4.49 (4.08–4.92)	4.31 (3.84–4.82)	3.60 (3.32–3.91)
Gastric or duodenal ulcer	4.00 (3.60–4.43)	3.80 (3.45–4.17)	6.47 (6.09–6.86)	6.68 (6.31–7.08)	5.98 (5.62–6.36)	5.20 (4.77–5.68)	3.97 (3.53–4.47)	4.95 (4.62–5.30)
Parkinson's disease	0.69 (0.53–0.89)	0.40 (0.29–0.53)	0.30 (0.22–0.41)	0.36 (0.28–0.47)	0.32 (0.24–0.43)	1.13 (0.93–1.37)	0.44 (0.31–0.64)	0.54 (0.44–0.68)
Bedsore	0.68 (0.52–0.88)	0.84 (0.68–1.03)	0.67 (0.55–0.81)	0.62 (0.51–0.76)	0.47 (0.37–0.59)	0.30 (0.21–0.44)	0.21 (0.12–0.36)	0.29 (0.22–0.40)

*Weight was calculated based on the age–sex–residence-specific distribution from the CLHLS study. Prevalence was presented as the rate (95% CI)*.

**Figure 4:**
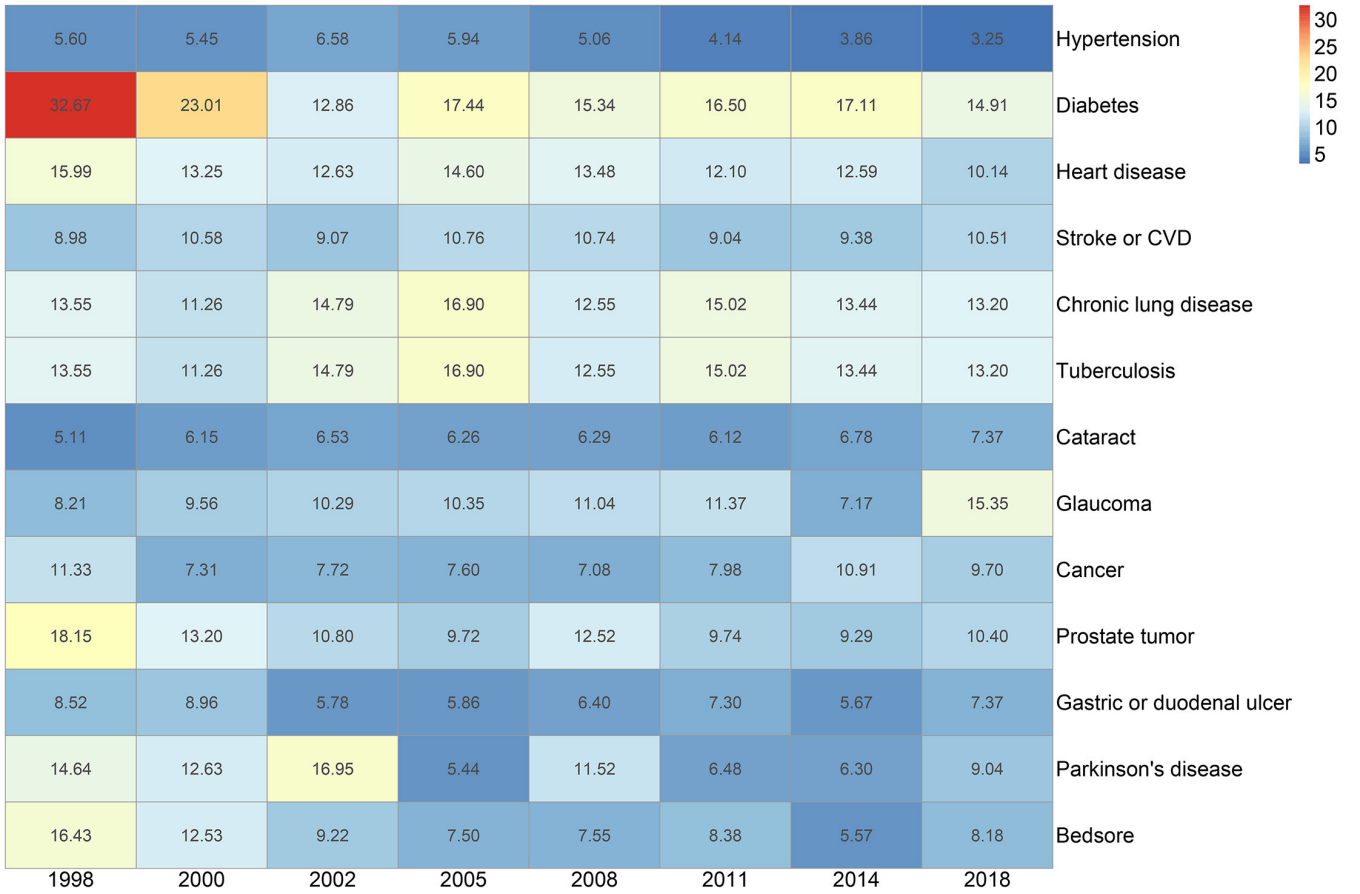
Heatmap of relative risk (RR) of singular chronic disease or condition by the survey of eight waves.

Figure 4 shows the RR values of single chronic disease or condition across the survey of eight waves. Among the 13 chronic conditions in this study, diabetes has the highest determined RR value. The RR of diabetes for the eight waves were 32.67, 23.01, 12.86, 17.44, 15.34, 16.50, 17.11, and 14.91, respectively, which meant that diabetes was more than 10 times likely to coexist with other chronic conditions. Apart from a decreasing trend of RR in diabetes, chronic conditions like stroke or CVD increased from 8.98% in 1998 to 10.51% in 2018 with the fluctuation over the 20 years.

**Figure 5:**
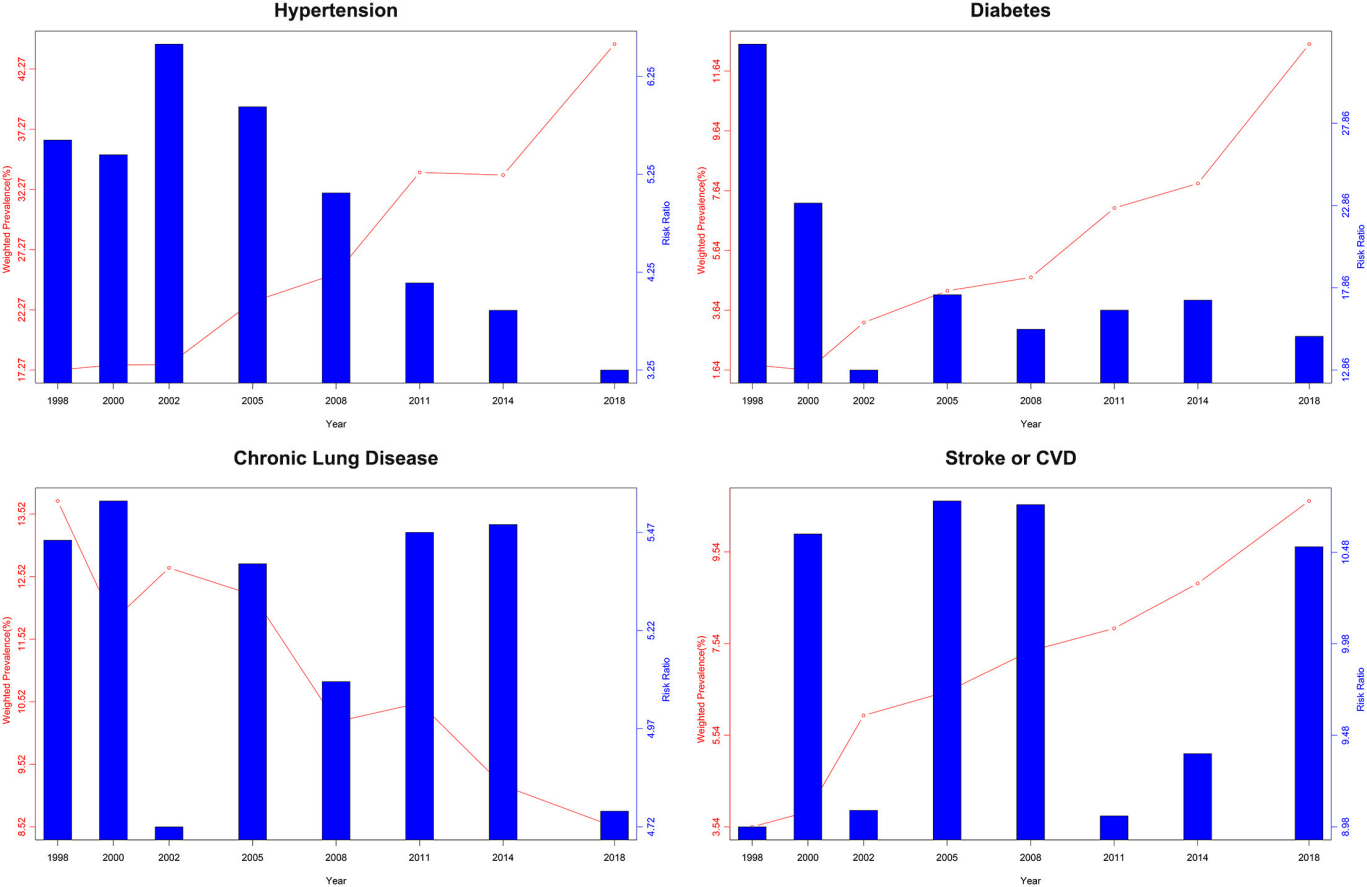
Weighted prevalence and RR of single chronic condition for the survey of eight waves.

The chronological change of prevalence and RR for some representative chronic conditions is displayed in Figure 5. There is no direct relationship between the RR value and the prevalence of the disease, judging from the inconsistency prevailing over the 20 years.

### Patterns of Multimorbidity

The five most prevalent and highest O/E multimorbidity combinations across the survey of eight waves are presented in Table 3. Hypertension appeared in three of the top 5 multimorbidity combinations during the 1998–2002 wave, rose to four of the top 5 multimorbidity combinations in the following three waves, and took up all the top multimorbidity combinations across the last two waves. Across the survey of eight waves, hypertension and heart disease, hypertension and cataract, and hypertension and chronic lung disease had been in the five most prevalent multimorbidity combinations. Hypertension and heart disease took the leading prevalence of the multimorbidity combinations, increasing from 2.34% in the 1998 wave to 9.82% in the 2018 wave (*p* = 0.002). A similar increasing trend was found in both the combination of hypertension and cataract and of hypertension and chronic lung disease (*p* = 0.035; *p* = 0.019). Chronic lung disease appeared prevalently in the first four survey waves and was replaced by a combination of hypertension and stroke or CVD.

**Table 3:** Prevalence and observed-to-expected ratio (O/E) of the five most common and prevalent multimorbidity combinations for the survey of eight waves.

**Wave**	**Morbidity dyads^a^**	**Prevalence in all**	**Prevalence in MCC**	**O/E ratio**	**Morbidity dyads^b^**	**Prevalence in all**	**Prevalence in MCC**	**O/E ratio**
1998	Hypertension + Cataract^*^	3.15	20.97	1.31	Cancer + Bedsore	0.09	0.59	19.43
	Chronic lung disease + Cataract	3.09	20.60	1.33	Tuberculosis + Cancer	0.07	0.44	14.57
	Heart disease + Cataract	2.79	18.62	2.05	Cancer + Parkinson's disease	0.07	0.44	13.22
	Hypertension + Heart disease^*^	2.34	15.62	2.49	Tuberculosis + Bedsore	0.08	0.51	10.46
	Hypertension + Chronic lung disease^*^	1.86	12.39	1.16	Glaucoma + Cancer	0.12	0.81	9.56
2000	Hypertension + Heart disease^*^	2.55	19.14	2.45	Cancer + Parkinson's disease	0.03	0.20	18.64
	Hypertension + Cataract^*^	2.39	17.94	1.34	Cancer + Bedsore	0.03	0.20	12.05
	Chronic lung disease + Cataract	1.88	14.12	1.31	Tuberculosis + Cancer	0.02	0.13	8.03
	Heart disease + Cataract	1.86	13.92	1.93	Cancer + Prostate tumor	0.05	0.40	7.01
	Hypertension + Chronic lung disease^*^	1.67	12.52	1.07	Glaucoma + Cancer	0.04	0.33	6.17
2002	Hypertension + Heart disease^*^	3.58	22.00	2.66	Cancer + Parkinson's disease	0.01	0.08	6.77
	Hypertension + Cataract^*^	2.49	15.30	1.37	Tuberculosis + Cancer	0.02	0.11	5.98
	Hypertension + Chronic lung disease^*^	2.30	14.15	1.17	Cancer + Prostate tumor	0.04	0.27	5.03
	Chronic lung disease + Cataract	2.27	13.96	1.46	Gastric or Duodenal ulcer + Parkinson's disease	0.11	0.65	4.60
	Heart disease + Cataract	1.93	11.86	1.82	Parkinson's disease + Bedsore	0.02	0.11	4.46
2005	Hypertension + Heart disease^*^	4.16	24.04	2.58	Cancer + Parkinson's disease	0.02	0.11	8.70
	Hypertension + Cataract^*^	3.10	17.91	1.42	Parkinson's disease + Bedsore	0.03	0.18	8.06
	Hypertension + Chronic lung disease^*^	2.68	15.47	1.25	Tuberculosis + Cancer	0.03	0.15	7.57
	Hypertension + Stroke or CVD	2.45	14.14	2.50	Cancer + Bedsore	0.03	0.15	7.09
	Chronic lung disease + Cataract	2.23	12.89	1.55	Tuberculosis + Bedsore	0.04	0.22	6.31
2008	Hypertension + Heart disease^*^	4.00	23.95	2.34	Tuberculosis + Cancer	0.03	0.18	10.37
	Hypertension + Cataract^*^	2.71	16.25	1.28	Parkinson's disease + Bedsore	0.02	0.11	6.36
	Hypertension + Stroke or CVD	2.39	14.31	2.07	Tuberculosis + Bedsore	0.02	0.11	5.02
	Hypertension + Chronic lung disease^*^	2.21	13.21	1.10	Cataract + Glaucoma	0.91	5.44	4.02
	Heart disease + Cataract	1.79	10.74	1.83	Tuberculosis + Prostate tumor	0.09	0.53	3.93
2011	Hypertension + Heart disease^*^	6.41	28.45	1.89	Tuberculosis + Bedsore	0.04	0.18	6.96
	Hypertension + Cataract^*^	4.36	19.36	1.31	Tuberculosis + Cancer	0.06	0.27	6.92
	Hypertension + Stroke or CVD	4.14	18.36	1.78	Cancer + Bedsore	0.03	0.14	6.42
	Hypertension + Chronic lung disease^*^	3.90	17.32	1.19	Cancer + Parkinson's disease	0.04	0.18	6.27
	Heart disease + Chronic lung disease	2.68	11.91	1.92	Glaucoma + Parkinson's disease	0.07	0.32	5.88
2014	Hypertension + Heart disease^*^	6.83	29.02	1.77	Parkinson's disease + Bedsore	0.06	0.24	16.11
	Hypertension + Stroke or CVD	4.76	20.21	1.81	Tuberculosis + Bedsore	0.03	0.12	10.82
	Hypertension + Cataract^*^	4.46	18.97	1.23	Tuberculosis + Cancer	0.04	0.18	10.11
	Hypertension + Chronic lung disease^*^	4.07	17.32	1.20	Cancer + Parkinson's disease	0.06	0.24	10.03
	Hypertension + Diabetes	3.31	14.07	2.00	Cancer + Bedsore	0.05	0.18	9.31
2018	Hypertension + Heart disease^*^	9.82	33.63	1.56	Tuberculosis + Bedsore	0.04	0.13	11.73
	Hypertension + Cataract^*^	6.60	22.61	1.30	Parkinson's disease + Bedsore	0.04	0.13	11.06
	Hypertension + Diabetes	6.55	22.43	1.85	Tuberculosis + Parkinson's disease	0.04	0.15	7.79
	Hypertension + Stroke or CVD	6.43	22.02	1.57	Glaucoma + Cancer	0.16	0.54	6.77
	Hypertension + Chronic lung disease^*^	3.96	13.55	1.02	Glaucoma + Parkinson's disease	0.09	0.30	6.32

a *The five most frequently occurring multimorbidity combinations*.

b *The five highest O/E multimorbidity combinations*.

* *p-trends for “hypertension + heart disease,” “hypertension + cataract,” and “hypertension + chronical lung disease” were 0.002, 0.035, and 0.019, respectively*.

*MCC, multiple chronic condition*.

As for the lists ranked by the value of O/E, cancer had a decreasing tendency to be the main component of the multimorbidity combinations, while bedsore, TB, and Parkinson's disease played an increasing role across the survey of eight waves. The highest O/E ratios of the multimorbidity combination in the 1998 wave were cancer and bedsore (19.43), and descended to 12.05 in 2000, 7.09 in 2005, 6.42 in 2011, and 9.31 in 2014. Cancer and Parkinson's disease took the leading O/E ratio in the period of 2000, 2002, and 2005 waves, the value of which were 18.64, 6.77, and 8.70, respectively. Of the survey of eight waves, TB and cancer went from 14.57 to 5.8, and increased to the highest O/E ratio (10.37) of all the combinations in 2008, remaining at 10.11 after the fluctuation in 2011. TB and bedsore had the highest O/E ratio in 2011 (6.96) and 2018 (11.73), respectively. The top O/E ratio of combinations in the 2014 wave was the Parkinson's disease cluster (16.11).

## Discussion

Based on the CLHLS survey data, this large-scale, national representative study provided unique spatiotemporal characteristics of the prevalence and pattern of multimorbidity among the older population in a temporal and spatial distribution. The results indicated that there is an increasing trend in the prevalence of multimorbidity among Chinese elderly from 1998 to 2018. Besides, multimorbidity was more prevalent in the northern than that in the southern part, and the gap became narrowing over time.

The prevalence of multimorbidity estimated in this study was 30.76% in the 2018 survey wave, which was relatively low compared with current researches. A recent study based on the 2011–2015 CHARLS data included 19,841 participants aged at least 50 years, and the prevalence of multimorbidity was 42.4% (16). In recent studies, 22.3–68.9% among the elderly reported had multimorbidity worldwide (13, 17, 19–21). We found huge differences from previous findings about the estimated prevalence of multimorbidity varying substantially, on account of the differences in study design, eligible diseases included, and various analytical methods (22). Existing studies assessing the distribution of the prevalence or pattern of multimorbidity in China are either frequently based on restricted population with limited data sources [geographic region (23, 24), administrative region (15, 25), and hospital admission (26, 27)], or referred to a cross-sectional study (28).

A chronological change of the prevalence of multimorbidity was observed to be consistent with several studies based on older population. An increasing trend of the prevalence of multimorbidity was observed in most European countries (29). Another longitudinal study including 6,593 older participants in Beijing (30) estimated the prevalence of multimorbidity following an upward trend over time. Also, the prevalence of multimorbidity across gender, age, and region-based categories was confirmed in our study. The reasons accounting for a chronological change might be associated with the long-standing disease burden corresponding to a rapid economic development as the public had access to medical resources and raised health awareness (31). The current status of high prevalence in multimorbidity among the elderly was mostly caused by the declining mortality rates of noncommunicable diseases combined with the evaluated life expectancy (32). A more interesting finding in this study was that single-disease prevalence had increased slightly, thus significantly rose the trend of multimorbidity. The results implied the improvement of diagnosis and treatment practices over time more than the rising global disease burden (33). Compared to the study conducted in Canada, a significant increase in the prevalence of multimorbidity attributed to obesity was identified (34). And, the pooled analysis of individuals from the US and European cohort showed the association between body mass index (BMI) and the risk of cardiometabolic multimorbidity (35). The rising trend could have an explanation by a rapid economic development over the past 20 years in China. It not only boosted the medical resource allocated to prolong life span, but also resulted in the great changes in health-related life styles of the public diet or other unhealthy behaviors. Our results set an alert to LMICs around the world, where government should account for more complex care needs and rising costs concerning multimorbidity among older adults.

The results indicated a significant geographic variability in the prevalence of multimorbidity over the 20 years. And, Shanghai took the leading prevalence of multimorbidity from 2002 to 2018 survey waves, showing an upward tendency significantly. Recent studies conducted in Shanghai reported the prevalence of multimorbidity, which were 49.2% in 2013 (36) and 74.3% in 2018 (37), respectively. Along with Beijing, the prevalence of multimorbidity in Shanghai represents the comprehensive utilization of healthcare and its increasing prevalence among the older population around the world (38, 39). Our results demonstrated that the majority of the surveyed provinces had an increasing trend across the eight waves. A sex-specific age–period–cohort study conducted in Hongkong showed (40) that there was an upward inflection of the risk of increased morbidity burden. As it is shown, the disparity between the north and the south became narrow over time (24, 41, 42). It urges the need to find out the mechanism how geographic characteristics impact on the progression of multimorbidity among the elderly. This is not only conducive to the etiologic research and clinical practice within local communities but also facilitates the policymakers of public health to allocate medical resources rationally (43).

Our study found that hypertension is relatively less likely to coexist with other chronic conditions or diseases. The risk ratios of hypertension were the lowest in all the diseases investigated, with a tendency decreasing from 5.6 to 3.25 over the 20 years. Moreover, hardly did hypertension appear in the five highest O/E value multimorbidity combinations across time. Even though the most common multimorbidity combinations incorporated a hypertension combination cluster, the prevalence of each disease (heart disease, cataract, and chronic lung disease) was more than 10% over time. The reason might be that a high prevalence of single disease contributes to the frequent occurrence of corresponding multimorbidity combinations, which was consistent with previous studies (17, 44). The trend in the prevalence of hypertension was in agreement with the current evidence, a study on hypertension among the oldest also reported that the prevalence had gradually increased over time (45), even though the awareness, treatment, and control rates of hypertension had made an impressive progress (46, 47). The prevalence of hypertension among the elderly was high in the reduced elasticity of large arteries, long-term build-up of plaque, and increased incidence of cardiac and vascular disease (48). Some studies suggested hormonal changes accounted for the result (49). There is an urgent importance to take measures to reducing hypertension to decrease the prevalence of corresponding morbidity combination.

Even though the prevalence of diabetes was relatively low compared with other surveyed diseases, diabetes was more likely to coexist with other diseases (the value of RR remained high over time). There is an indication of the gap between a high prevalence and the possibility of coexistence with other diseases (50, 51). A study identified diabetes-multimorbidity combinations among the older adults (52), adding to the understanding of an intact relationship between diabetes and other diseases. Another study comprising UK and Taiwan community cohorts identified the association between multimorbidity, T2D, and mortality (53). These results were in line with the current studies. And there might be some explanations accounting for the associations: the elderly with diabetes received regular or better quality of care and health service, resulting in more chances of the early diagnosis of other diseases (54). In addition to higher utilization, survival bias might also play an important role in the longitudinal investigation assessment, and hence achieving a higher coexistence with multimorbidity. Moreover, the association between depression and diabetes was significantly proven in current studies (55, 56), better knowledge of the pathogen mechanisms needing to identify allows a more effective tailored care.

Besides, the RR for cancer, bedsore, TB, and Parkinson's disease was also relatively high and in accordance with the frequent occurrences in the top 5 multimorbidity combination ranked by O/E over time. Previous studies showed that in elderly adults the common multimorbidity patterns contain: the vascular-metabolic cluster, the stomach-arthritis cluster, the cognitive-emotional cluster, and the hepatorenal cluster, varying by gender and residential regions (16, 57). While our study found out that the patterns of cancer and Parkinson's disease, cancer and TB, and TB and bedsore were the frequent binary multimorbidity combinations over time. These clusters may share the common risk factors, like poverty or other socioeconomic status. The gap was derived from the difference of disease lists, regions, and analytical methods. Mapping these clusters using the longitudinal data could help to identify many more links between diseases or conditions, which is crucial to uncover new mechanisms for diseases, to develop treatments, and to reconfigure services to better meet the patient's need (58). These results indicate further etiological study, the generation of prevention strategies, and the formulation of public health policies.

To the best of our knowledge, this is the first study to describe the temporal and spatial trends in the prevalence of multimorbidity and to explore chronological changes of comorbidity patterns in a large, representative, older population survey in China. However, our study had several limitations. Firstly, CLHLS covered 23 provinces of China over the 20 years and represented the elderly well in China, but the data for the unselected provinces were lacking. Even though we analyzed the prevalence by weighting to account for the complex, multistage design of the study, and non-response in the data, the global representativeness and generalizability of the results are limited. Secondly, the CLHLS questionnaire did not investigate all the chronic diseases or conditions typically included in clinical database studies, and only 13 chronic diseases or conditions were included in the analysis to show the chronological trend of multimorbidity as long as possible. Lastly, data in this study was investigated based on self-reporting. Although the quality of data was ensured with an elaborate design and considerate control, it could cause unavoidable information bias and the overestimated prevalence due to the increasing diagnosis and awareness. We expect further studies including a wider range of chronic diseases and robust measures of psychological and physical disorders, and make advances in data science helping to uncover the linkages and correlations among the clusters to capture multimorbidity across the health system.

## Conclusion

Our findings indicated that high attention should be paid to the longstanding issue of multimorbidity among the elderly in the context of aging population, and the observed geographic disparities need to be investigated profoundly for macroscopic policy regulation and formulation. Policymakers in LMICs have global implications: a rapid economic growth along with the acceleration of the aging population brought new challenges associated with multimorbidity, urging the healthcare resource planning and the generation of prevention strategies.

## Data Availability Statement

Publicly available datasets were analyzed in this study. This data can be found at: https://opendata.pku.edu.cn/dataset.xhtml?persistentId=10.18170/DVN/WBO7LK.

## Ethics Statement

The studies involving human participants were reviewed and approved by Biomedical Ethics Committee of Peking University. The patients/participants provided their written informed consent to participate in this study.

## Author Contributions

SC and ML conceived and designed the study, carried out the data analysis, and wrote the report. SC, SW, WJ, KH, and YS contributed to acquiring the data. ML and YH supervised the study. All authors contributed to analyzing the data, interpreting the results, drafting the manuscript, and the revisions.

## Funding

This study was supported by National Natural Science Foundation of China (Nos. 81773502, 82173589, and 82173590).

## Conflict of Interest

The authors declare that the research was conducted in the absence of any commercial or financial relationships that could be construed as a potential conflict of interest.

## Publisher's Note

All claims expressed in this article are solely those of the authors and do not necessarily represent those of their affiliated organizations, or those of the publisher, the editors and the reviewers. Any product that may be evaluated in this article, or claim that may be made by its manufacturer, is not guaranteed or endorsed by the publisher.

## References

[1] Yarnall AJ Sayer AA Clegg A Rockwood K Parker S Hindle JV. New horizons in multimorbidity in older adults. Age Ageing. (2017) 46:882–8. 10.1093/ageing/afx150 28985248 PMC5860018

[2] Dugravot A Fayosse A Dumurgier J Bouillon K Rayana TB Schnitzler A. Social inequalities in multimorbidity, frailty, disability, and transitions to mortality: a 24-year follow-up of the Whitehall II cohort study. Lancet Public Health. (2020) 5:e42–50. 10.1016/S2468-2667(19)30226-9 31837974 PMC7098476

[3] Makovski TT Schmitz S Zeegers MP Stranges S van den Akker M. Multimorbidity and quality of life: systematic literature review and meta-analysis. Ageing Res Rev. (2019) 53:100903. 10.1016/j.arr.2019.04.005 31048032

[4] Vetrano DL Palmer K Marengoni A Marzetti E Lattanzio F Roller-Wirnsberger R. Frailty and multimorbidity: a systematic review and meta-analysis. J Gerontol Ser A Biol Sci Med Sci. (2019) 74:659–66. 10.1093/gerona/gly110 29726918

[5] Kadambi S Abdallah M Loh KP. Multimorbidity, function, and cognition in aging. Clin Geriatr Med. (2020) 36:569–84. 10.1016/j.cger.2020.06.002 33010895 PMC8012012

[6] Hajek A Kretzler B König HH. Multimorbidity, loneliness, and social isolation. A systematic review. Int J Environ Res Public Health. (2020) 17:228688. 10.3390/ijerph17228688 33238506 PMC7700324

[7] Barnett K Mercer SW Norbury M Watt G Wyke S Guthrie B. Epidemiology of multimorbidity and implications for health care, research, and medical education: a cross-sectional study. Lancet. (2012) 380:37–43. 10.1016/S0140-6736(12)60240-2 22579043

[8] Low LL Kwan YH Ko MSM Yeam CT Lee VSY Tan WB. Epidemiologic characteristics of multimorbidity and sociodemographic factors associated with multimorbidity in a rapidly aging Asian Country. J Am Med Assoc Netw Open. (2019) 2:e1915245. 10.1001/jamanetworkopen.2019.15245 31722030 PMC6902794

[9] Pearson-Stuttard J Ezzati M Gregg EW. Multimorbidity-a defining challenge for health systems. Lancet Public Health. (2019) 4:e599–600. 10.1016/S2468-2667(19)30222-1 31812234

[10] Desdín-Micó G Soto-Heredero G Aranda JF Oller J Carrasco E Gabandé-Rodríguez E. T cells with dysfunctional mitochondria induce multimorbidity and premature senescence. Science. (2020) 368:1371–6. 10.1126/science.aax0860 32439659 PMC7616968

[11] Foscolou A Chrysohoou C Dimitriadis K Masoura K Vogiatzi G Gkotzamanis V. The association of healthy aging with multimorbidity: IKARIA study. Nutrients. (2021) 13:41386. 10.3390/nu13041386 33924100 PMC8074281

[12] Nguyen H Wu YT Dregan A Vitoratou S Chua KC Prina AM. Multimorbidity patterns, all-cause mortality and healthy aging in older English adults: results from the English Longitudinal Study of Aging. Geriatr Gerontol Int. (2020) 20:1126–32. 10.1111/ggi.14051 33030261

[13] Zhao Y Atun R Oldenburg B McPake B Tang S Mercer SW. Physical multimorbidity, health service use, and catastrophic health expenditure by socioeconomic groups in China: an analysis of population-based panel data. Lancet Global Health. (2020) 8:e840–e9. 10.1016/S2214-109X(20)30127-3 32446349 PMC7241981

[14] Fang EF Scheibye-Knudsen M Jahn HJ Li J Ling L Guo H. A research agenda for aging in China in the 21st century. Ageing Res Rev. (2015) 24:197–205. 10.1016/j.arr.2015.08.003 26304837 PMC5179143

[15] Chen H Cheng M Zhuang Y Broad JB. Multimorbidity among middle-aged and older persons in urban China: prevalence, characteristics and health service utilization. Geriatr Gerontol Int. (2018) 18:1447–52. 10.1111/ggi.13510 30178629

[16] Yao SS Cao GY Han L Chen ZS Huang ZT Gong P. Prevalence and patterns of multimorbidity in a nationally representative sample of older Chinese: results from the China health and retirement longitudinal study. J Gerontol Ser A Biol Sci Med Sci. (2020) 75:1974–80. 10.1093/gerona/glz185 31406983

[17] Zhang R Lu Y Shi L Zhang S Chang F. Prevalence and patterns of multimorbidity among the elderly in China: a cross-sectional study using national survey data. BMJ Open. (2019) 9:e024268. 10.1136/bmjopen-2018-024268 31427309 PMC6701688

[18] Studies CfHAaD. The Chinese Longitudinal Healthy Longevity Survey (CLHLS)-Longitudinal Data (1998–2018). Beijing: Peking University Open Research Data Platform, DRAFT VERSION. (2020).

[19] Kyprianidou M Panagiotakos D Faka A Kambanaros M Makris KC Christophi CA. Prevalence of multimorbidity in the Cypriot population; a cross-sectional study (2018–2019). PLoS ONE. (2020) 15:e0239835. 10.1371/journal.pone.0239835 33104700 PMC7588119

[20] Cassell A Edwards D Harshfield A Rhodes K Brimicombe J Payne R. The epidemiology of multimorbidity in primary care: a retrospective cohort study. Br J Gen Pract. (2018) 68:e245–e51. 10.3399/bjgp18X695465 29530918 PMC5863678

[21] Zemedikun DT Gray LJ Khunti K Davies MJ Dhalwani NN. Patterns of multimorbidity in middle-aged and older adults: an analysis of the UK Biobank data. Mayo Clin Proc. (2018) 93:857–66. 10.1016/j.mayocp.2018.02.012 29801777

[22] Ofori-Asenso R Chin KL Curtis AJ Zomer E Zoungas S Liew D. Recent patterns of multimorbidity among older adults in high-income countries. Popul Health Manag. (2019) 22:127–37. 10.1089/pop.2018.0069 30096023

[23] You L Yu Z Zhang X Wu M Lin S Zhu Y. Association between multimorbidity and depressive symptom among community-dwelling elders in Eastern China. Clin Interv Aging. (2019) 14:2273–80. 10.2147/CIA.S221917 31908437 PMC6929925

[24] Wang HH Wang JJ Wong SY Wong MC Li FJ Wang PX. Epidemiology of multimorbidity in China and implications for the healthcare system: cross-sectional survey among 162,464 community household residents in southern China. BMC Med. (2014) 12:188. 10.1186/s12916-014-0188-0 25338506 PMC4212117

[25] Gu J Chao J Chen W Xu H Zhang R He T. Multimorbidity and health-related quality of life among the community-dwelling elderly: a longitudinal study. Arch Gerontol Geriatr. (2018) 74:133–40. 10.1016/j.archger.2017.10.019 29096228

[26] Lai FTT Wong SYS Yip BHK Guthrie B Mercer SW Chung RY. Multimorbidity in middle age predicts more subsequent hospital admissions than in older age: a nine-year retrospective cohort study of 121,188 discharged in-patients. Eur J Intern Med. (2019) 61:103–11. 10.1016/j.ejim.2018.12.001 30581041

[27] Tromp J Tay WT Ouwerkerk W Teng TK Yap J MacDonald MR. Multimorbidity in patients with heart failure from 11 Asian regions: a prospective cohort study using the ASIAN-HF registry. PLoS Med. (2018) 15:e1002541. 10.1371/journal.pmed.1002541 29584721 PMC5870945

[28] Lu J Wang Y Hou L Zuo Z Zhang N Wei A. Multimorbidity patterns in old adults and their associated multi-layered factors: a cross-sectional study. BMC Geriatr. (2021) 21:372. 10.1186/s12877-021-02292-w 34147073 PMC8214251

[29] van den Akker M Vaes B Goderis G Van Pottelbergh G De Burghgraeve T Henrard S. Trends in multimorbidity and polypharmacy in the Flemish-Belgian population between 2000 and 2015. PLoS One. (2019) 14:e0212046. 10.1371/journal.pone.0212046 30753214 PMC6372187

[30] ZhangLSunFLiYTangZMaL. Multimorbidity in community-dwelling older adults in Beijing: prevalence and trends, 2004–2017. J Nutr Health Aging. (2021) 25:116–9. 10.1007/s12603-020-1467-433367471

[31] Pefoyo AJ Bronskill SE Gruneir A Calzavara A Thavorn K Petrosyan Y. The increasing burden and complexity of multimorbidity. BMC Public Health. (2015) 15:415. 10.1186/s12889-015-1733-2 25903064 PMC4415224

[32] McGrail K Lavergne R Lewis S. The chronic disease explosion: artificial bang or empirical whimper? Bmj. (2016) 352:i1312. 10.1136/bmj.i1312 26980185

[33] Feely A Lix LM Reimer K. Estimating multimorbidity prevalence with the Canadian Chronic Disease Surveillance System. Health Promot Chronic Dis Prev Can. (2017) 37:215–22. 10.24095/hpcdp.37.7.02 28703703 PMC5650032

[34] Lebenbaum M Zaric GS Thind A Sarma S. Trends in obesity and multimorbidity in Canada. Prev Med. (2018) 116:173–9. 10.1016/j.ypmed.2018.08.025 30194961

[35] Kivimäki M Kuosma E Ferrie JE Luukkonen R Nyberg ST Alfredsson L. Overweight, obesity, and risk of cardiometabolic multimorbidity: pooled analysis of individual-level data for 120 813 adults from 16 cohort studies from the USA and Europe. Lancet Public Health. (2017) 2:e277–e85. 10.1016/S2468-2667(17)30074-9 28626830 PMC5463032

[36] Su P Ding H Zhang W Duan G Yang Y Chen R. The association of multimorbidity and disability in a community-based sample of elderly aged 80 or older in Shanghai, China. BMC Geriatr. (2016) 16:178. 10.1186/s12877-016-0352-9 27784269 PMC5081877

[37] Wang Z Peng W Li M Li X Yang T Li C. Association between multimorbidity patterns and disability among older people covered by long-term care insurance in Shanghai, China. BMC Public Health. (2021) 21:418. 10.1186/s12889-021-10463-y 33639902 PMC7912511

[38] Chudasama YV Khunti KK Zaccardi F Rowlands AV Yates T Gillies CL. Physical activity, multimorbidity, and life expectancy: a UK Biobank longitudinal study. BMC Med. (2019) 17:108. 10.1186/s12916-019-1339-0 31186007 PMC6560907

[39] Garin N Koyanagi A Chatterji S Tyrovolas S Olaya B Leonardi M. Global multimorbidity patterns: a cross-sectional, population-based, multi-country study. J Gerontol Ser A Biol Sci Med Sci. (2016) 71:205–14. 10.1093/gerona/glv128 26419978 PMC5864156

[40] Lai FTT Guthrie B Wong SYS Yip BHK Chung GKK Yeoh EK. Sex-specific intergenerational trends in morbidity burden and multimorbidity status in Hong Kong community: an age-period-cohort analysis of repeated population surveys. BMJ Open. (2019) 9:e023927. 10.1136/bmjopen-2018-023927 30782718 PMC6347870

[41] Bao XY Xie YX Zhang XX Peng X Huang JX Du QF. The association between multimorbidity and health-related quality of life: a cross-sectional survey among community middle-aged and elderly residents in southern China. Health Qual Life Outcomes. (2019) 17:107. 10.1186/s12955-019-1175-0 31234889 PMC6591935

[42] Peng X Bao X Xie Y Zhang X Huang J Liu Y. The mediating effect of pain on the association between multimorbidity and disability and impaired physical performance among community-dwelling older adults in southern China. Aging Clin Exp Res. (2020) 32:1327–34. 10.1007/s40520-019-01324-1 31522389

[43] Cromley EK Wilson-Genderson M Heid AR Pruchno RA. Spatial associations of multiple chronic conditions among older adults. J Appl Gerontol. (2018) 37:1411–35. 10.1177/0733464816672044 27697796

[44] Afshar S Roderick PJ Kowal P Dimitrov BD Hill AG. Multimorbidity and the inequalities of global ageing: a cross-sectional study of 28 countries using the World Health Surveys. BMC Public Health. (2015) 15:776. 10.1186/s12889-015-2008-7 26268536 PMC4534141

[45] Du J Zhu G Yue Y Liu M He Y. Blood pressure and hypertension prevalence among oldest-old in China for 16 year: based on CLHLS. BMC Geriatr. (2019) 19:248. 10.1186/s12877-019-1262-4 31500574 PMC6734230

[46] Wang Z Chen Z Zhang L Wang X Hao G Zhang Z. Status of hypertension in China: results from the China Hypertension Survey, 2012–2015. Circulation. (2018) 137:2344–56. 10.1161/CIRCULATIONAHA.117.032380 29449338

[47] Mills KT Stefanescu A He J. The global epidemiology of hypertension. Nat Rev Nephrol. (2020) 16:223–37. 10.1038/s41581-019-0244-2 32024986 PMC7998524

[48] DeGuire J Clarke J Rouleau K Roy J Bushnik T. Blood pressure and hypertension. Health Rep. (2019) 30:14–21. 30785635

[49] Huang XB Zhang Y Wang TD Liu JX Yi YJ Liu Y. Prevalence, awareness, treatment, and control of hypertension in southwestern China. Sci Rep. (2019) 9:19098. 10.1038/s41598-019-55438-7 31836764 PMC6911047

[50] Zhang J Yu KF. What's the relative risk? A method of correcting the odds ratio in cohort studies of common outcomes. J Am Med Assoc. (1998) 280:1690–1. 10.1001/jama.280.19.1690 9832001

[51] Noordzij M van Diepen M Caskey FC Jager KJ. Relative risk versus absolute risk: one cannot be interpreted without the other. Nephrol Dial Transplant. (2017) 32(suppl_2):ii13–8. 10.1093/ndt/gfw465 28339913

[52] Quiñones AR Markwardt S Botoseneanu A. Diabetes-multimorbidity combinations and disability among middle-aged and older adults. J Gen Intern Med. (2019) 34:944–51. 10.1007/s11606-019-04896-w 30815788 PMC6544693

[53] Chiang JI Hanlon P Li TC Jani BD Manski-Nankervis JA Furler J. Multimorbidity, mortality, and HbA1c in type 2 diabetes: a cohort study with UK and Taiwanese cohorts. PLoS Med. (2020) 17:e1003094. 10.1371/journal.pmed.1003094 32379755 PMC7205223

[54] Freisling H Viallon V Lennon H Bagnardi V Ricci C Butterworth AS. Lifestyle factors and risk of multimorbidity of cancer and cardiometabolic diseases: a multinational cohort study. BMC Med. (2020) 18:5. 10.1186/s12916-019-1474-7 31918762 PMC6953215

[55] McClellan SP Haque K García-Peña C. Diabetes multimorbidity combinations and disability in the Mexican Health and Aging Study, 2012–2015. Arch Gerontol Geriatr. (2021) 93:104292. 10.1016/j.archger.2020.104292 33186887 PMC7887040

[56] Ouk M Wu CY Colby-Milley J Fang J Zhou L Shah BR. Depression and diabetes mellitus multimorbidity is associated with loss of independence and dementia poststroke. Stroke. (2020) 51:3531–40. 10.1161/STROKEAHA.120.031068 33226916

[57] Nguyen QD Wu C Odden MC Kim DH. Multimorbidity patterns, frailty, and survival in community-dwelling older adults. J Gerontol Ser A Biol Sci Med Sci. (2019) 74:1265–70. 10.1093/gerona/gly205 30169580 PMC6625582

[58] Whitty CJM Watt FM. Map clusters of diseases to tackle multimorbidity. Nature. (2020) 579:494–6. 10.1038/d41586-020-00837-4 32210388

