# Supplementary material for: Spatiotemporal Analysis of the Prevalence and Pattern of Multimorbidity in Older Chinese Adults
Source: Front Med (Lausanne). 2022 Jan 20;8:806616. doi: 10.3389/fmed.2021.806616 (PMC8811186; doi:10.3389/fmed.2021.806616)
Supplement: Supplementary file 1 [file Data_Sheet_1.docx]

**Supplementary Appendix**

Supplementary Table 1 Demographic characteristics of 8 survey waves

Supplementary Table 2 Chronic diseases or conditions list for the 8 survey waves

Supplementary Table 3 Prevalence of multimorbidity by gender and age in the 8 survey waves(%)

Supplementary Table 4 Prevalence of multimorbidity by province across the 8 survey waves (%)

Supplementary Table 5 Crude prevalence and RR of singular chronic disease or condition by 8 survey waves

Supplementary Table 1 Demographic characteristics of 8 survey waves

| Wave | 1998 | 2000 | 2002 | 2005 | 2008 | 2011 | 2014 | 2018 |
| --- | --- | --- | --- | --- | --- | --- | --- | --- |
| Total | 9,093 | 11,199 | 16,064 | 15,638 | 16,954 | 9,765 | 7,192 | 15,874 |
| Age (M, Q) | 92(85,100) | 91(85,98) | 87(77,96) | 87(76,95) | 89(78,96) | 86(76,94) | 85(77,93) | 85(76,95) |
| Catalog of age  ~80  81~90  91~100  101~ | 563(6.19)  3,471(38.17)  3,555(39.10)  1,504(16.54) | 621(5.55)  4,944(44.15)  3,891(34.74)  1,743(15.56) | 5,208(32.42)  4,372(27.22)  4,193(26.10)  2,291(14.26) | 5,280(33.76)  4,230(27.05)  4,050(25.90)  2,078(13.29) | 5,131(30.26)  4,554(26.86)  4,732(27.91)  2,537(14.97) | 3,463(35.46)  2,655(27.19)  2,410(24.68)  1,237(12.67) | 2,696(37.48)  2,184(30.37)  1,618(22.50)  694(9.65) | 5,919(37.29)  4,133(26.04)  3,804(23.96)  2,018(12.71) |
| Gender  Male  Female | 3,639(40.02)  5,454(59.98) | 4,651(41.53)  6,548(58.47) | 6,845(42.61)  9,219(57.39) | 6,688(42.77)  8,950(57.23) | 7,252(42.77)  9,702(57.23) | 4,398(45.04)  5,367(54.96) | 3,316(46.11)  3,876(53.89) | 6,925(43.62)  8,949(56.38) |
| Residence  City  Town  Rural | 3,469(38.15)  -  5,624(61.85) | 3,387(30.24)  3,512(31.36)  4,300(38.40) | 3,845(23.94)  3,549(22.09)  8,670(53.97) | 3,879(24.80)  3,101(19.83)  8,658(55.37) | 3,351(19.77)  3,310(19.52)  10,293(60.71) | 1,735(17.77)  2,885(29.54)  5,145(52.69) | 988(13.74)  2,224(30.92)  3,980(55.34) | 3,542(22.31)  5,238(33.00)  7,094(44.69) |

-: The survey conducted in 1998 didn’t subdivide the residence group into city and town.

Continuous variables and categoric variables were presented as median (quartile) and count (percentage), respectively.

Supplementary Table 2 Chronic diseases or conditions list for the 8 survey waves

| Wave | Chronic disease or conditions | Count |
| --- | --- | --- |
| 1998 | Hypertension, Diabetes, Heart disease, Stroke or CVD, Chronic lung disease (bronchitis, emphysema, pneumonia, asthma), Tuberculosis, Cataract, Glaucoma, Cancer, Prostate tumor, Gastric or duodenal ulcer, Parkinson’s disease, Bedsore | 13 |
| 2000 | Hypertension, Diabetes, Heart disease, Stroke or CVD, Chronic lung disease (bronchitis, emphysema, pneumonia, asthma), Tuberculosis, Cataract, Glaucoma, Cancer, Prostate tumor, Gastric or duodenal ulcer, Parkinson’s disease, Bedsore, Arthritis, Dementia | 15 |
| 2002 | Hypertension, Diabetes, Heart disease, Stroke or CVD, Chronic lung disease (bronchitis, emphysema, pneumonia, asthma), Tuberculosis, Cataract, Glaucoma, Cancer, Prostate tumor, Gastric or duodenal ulcer, Parkinson’s disease, Bedsore, Arthritis, Dementia, Mental disease, Orthopedic disease, Internal medical disease, Dermatosis, Five organs disease, Gynecological disease | 21 |
| 2005 | Hypertension, Diabetes, Heart disease, Stroke or CVD, Chronic lung disease (bronchitis, emphysema, pneumonia, asthma), Tuberculosis, Cataract, Glaucoma, Cancer, Prostate tumor, Gastric or duodenal ulcer, Parkinson’s disease, Bedsore, Arthritis, Dementia, Psychosis, Orthopedic disease, Internal medical disease, Dermatosis, Five organs disease, Gynecological disease | 21 |
| 2008 | Hypertension, Diabetes, Heart disease, Stroke or CVD, Chronic lung disease (bronchitis, emphysema, pneumonia, asthma), Tuberculosis, Cataract, Glaucoma, Cancer, Prostate tumor, Gastric or duodenal ulcer, Parkinson’s disease, Bedsore, Arthritis, Dementia, Epilepsy, Cholecystitis (cholelith disease), Blood disease, Chronic nephritis, Galactophore disease, Uterine tumor, Hepatitis | 22 |
| 2011 | Hypertension, Diabetes, Heart disease, Stroke or CVD, Chronic lung disease (bronchitis, emphysema, pneumonia, asthma), Tuberculosis, Cataract, Glaucoma, Cancer, Prostate tumor, Gastric or duodenal ulcer, Parkinson’s disease, Bedsore, Arthritis, Dementia, Epilepsy, Cholecystitis (cholelith disease), Blood disease, Rheumatism or rheumatoid disease, Chronic nephritis, Galactophore disease, Uterine tumor, Hyperplasia of prostate gland, Hepatitis | 24 |
| 2014 | Hypertension, Diabetes, Heart disease, Stroke or CVD, Chronic lung disease (bronchitis, emphysema, pneumonia, asthma), Tuberculosis, Cataract, Glaucoma, Cancer, Prostate tumor, Gastric or duodenal ulcer, Parkinson’s disease, Bedsore, Arthritis, Dementia, Epilepsy, Cholecystitis (cholelith disease), Blood disease, Rheumatism or rheumatoid disease, Chronic nephritis, Galactophore disease, Uterine tumor, Hyperplasia of prostate gland, Hepatitis | 24 |
| 2018 | Hypertension, Diabetes, Heart disease, Stroke or CVD, Chronic lung disease (bronchitis, emphysema, pneumonia, asthma), Tuberculosis, Cataract, Glaucoma, Cancer, Prostate tumor, Gastric or duodenal ulcer, Parkinson’s disease, Bedsore, Arthritis, Dementia, Epilepsy, Cholecystitis (cholelith disease), Dyslipidemia, Rheumatism or rheumatoid disease, Chronic nephritis, mammary gland hyperplasia, Uterine tumor, Prostatic hyperplasia, Hepatitis | 24 |

Supplementary Table 3 Prevalence of multimorbidity by gender and age in the 8 survey waves(%)

| Gender | Age | 1998 | 2000 | 2002 | 2005 | 2008 | 2011 | 2014 | 2018 | P for trend | Trend |
| --- | --- | --- | --- | --- | --- | --- | --- | --- | --- | --- | --- |
| Male | ~80 | 22.03 | 18.68 | 17.79 | 20.65 | 17.41 | 24.76 | 25.17 | 29.91 | 0.174 | 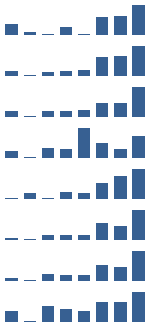 |
|  | 81~90 | 18.37 | 15.81 | 18.21 | 18.73 | 19.05 | 26.34 | 27.02 | 32.96 | 0.004 |  |
|  | 91~100 | 16.38 | 13.27 | 16.55 | 16.34 | 16.7 | 20.79 | 20.99 | 29.59 | 0.009 |  |
|  | 101~ | 13.17 | 10.86 | 14.07 | 13.83 | 20.95 | 15.88 | 13.71 | 18.24 | 0.174 |  |
| Female | ~80 | 16.97 | 20.51 | 17.39 | 20.68 | 20.27 | 26.19 | 30.71 | 34.58 | 0.009 |  |
|  | 81~90 | 14.79 | 13.84 | 16.93 | 17.14 | 17.3 | 25.56 | 23.26 | 34.84 | 0.004 |  |
|  | 91~100 | 12.51 | 11.28 | 13.98 | 13.68 | 13.49 | 17.61 | 17.11 | 23.64 | 0.035 |  |
|  | 101~ | 11.86 | 9.61 | 13.01 | 12.43 | 12.02 | 13.84 | 13.86 | 16.11 | 0.018 |  |

The variation across time was estimated by Mann-Kendall Trend Test.

Supplementary Table 4 Prevalence of multimorbidity by province across the 8 survey waves

| Province | 1998 | 2000 | 2002 | 2005 | 2008 | 2011 | 2014 | 2018 | P for trend | Trend |
| --- | --- | --- | --- | --- | --- | --- | --- | --- | --- | --- |
| Beijing | 87(76.99) | 73(32.3) | 84(26.01) | 95(28.27) | 111(36.88) | 63(40.65) | 39(43.82) | 356(57.23) | 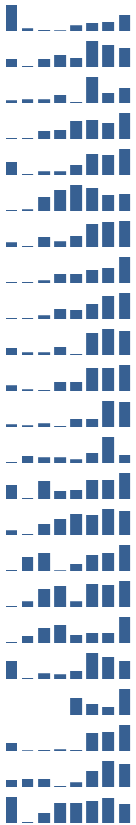0.266 |  |
| Tianjin | 28(27.45) | 23(19.01) | 55(27.36) | 62(31.16) | 36(28.57) | 24(46.15) | 14(42.42) | 36(38.71) | 0.063 |  |
| Hebei | 10(14.29) | 19(15.08) | 28(15.82) | 39(21.2) | 16(10.32) | 34(47.89) | 11(25) | 38(31.15) | 0.063 |  |
| Shanxi | 7(9.72) | 11(9.17) | 35(19.66) | 37(20.79) | 49(31.41) | 25(33.33) | 13(28.89) | 44(42.31) | 0.009 |  |
| Liaoning | 82(26.89) | 65(14.87) | 132(18.8) | 124(18.08) | 144(23.96) | 105(34.54) | 53(33.13) | 139(39.15) | 0.063 |  |
| Jilin | 19(12.67) | 39(14.44) | 108(24.88) | 136(31.26) | 117(35.67) | 55(32.74) | 25(26.88) | 46(27.54) | 0.108 |  |
| Heilongjiang | 36(25.17) | 51(19.92) | 116(31.61) | 102(27.13) | 107(32.62) | 57(46.34) | 39(49.37) | 91(50) | 0.004 |  |
| Shanghai | 107(26.68) | 119(26.21) | 204(32.43) | 282(43.38) | 228(43.68) | 78(52.7) | 32(57.14) | 508(79.5) | 0.002 |  |
| Jiangsu | 115(9.87) | 124(9.27) | 259(12.59) | 322(17.43) | 227(17.13) | 214(21.97) | 230(29.04) | 629(31.99) | 0.004 |  |
| Zhejiang | 121(16.37) | 107(11.36) | 177(11.37) | 216(16.59) | 91(8.59) | 200(32.95) | 147(36.93) | 209(34.95) | 0.108 |  |
| Anhui | 69(13.83) | 68(11.41) | 77(9.73) | 134(16.6) | 127(16.14) | 96(26.97) | 55(27.09) | 175(29.26) | 0.019 |  |
| Fujian | 62(15.27) | 74(14.8) | 89(16.12) | 64(13.79) | 59(18.1) | 18(18.37) | 19(28.36) | 60(27.91) | 0.035 |  |
| Jiangxi | 16(11.03) | 35(16.75) | 48(15.89) | 50(15.67) | 39(13.98) | 30(18.87) | 34(32.08) | 73(17.42) | 0.174 |  |
| Shandong | 68(16.50) | 51(10.78) | 136(18.23) | 99(14.18) | 302(14.21) | 258(18.68) | 217(18.53) | 423(21.44) | 0.063 |  |
| Henan | 33(9.35) | 28(6.09) | 84(13.35) | 119(16.93) | 248(20.5) | 193(20.02) | 168(23.97) | 265(22.69) | 0.009 |  |
| Hubei | 27(8.85) | 86(20.19) | 152(23.6) | 52(9.49) | 108(14.67) | 98(22.07) | 93(23.48) | 193(30.49) | 0.063 |  |
| Hunan | 18(5.81) | 36(9.4) | 114(17.04) | 125(19.03) | 85(9.6) | 92(20.81) | 91(20.13) | 181(22.46) | 0.009 |  |
| Guangdong | 79(11.43) | 124(17.06) | 193(24.71) | 198(27.05) | 169(18.63) | 108(20.42) | 83(20.05) | 327(34.49) | 0.108 |  |
| Guangxi | 142(11.33) | 110(8.09) | 180(9.09) | 180(8.91) | 196(9.51) | 153(12.74) | 107(11.93) | 211(11.29) | 0.266 |  |
| Hainan | - | - | - | - | 61(14.73) | 35(9.8) | 19(6.74) | 78(23.21) | 1.000 |  |
| Chongqing | 69(22.04) | 72(18.09) | 93(17.75) | 102(18.31) | 111(17.54) | 88(28.39) | 56(29.17) | 171(33.46) | 0.174 |  |
| Sichuan | 111(11.17) | 143(12.24) | 182(11.74) | 96(5.59) | 144(9.47) | 138(18.72) | 118(26.58) | 328(23.75) | 0.174 |  |
| Shaanxi | 58(38.16) | 36(17.14) | 68(25.28) | 74(33.04) | 56(33.14) | 38(34.55) | 29(37.18) | 55(32.54) | 0.536 |  |

Data was presented as count (percentage)

Mann-Kendall Trend Test was conducted to test the variation of multimorbidity across time.

Supplementary Table 5 Crude prevalence and RR of singular chronic disease or condition by 8 survey waves

| Wave | 1998 | 2000 | 2002 | 2005 | 2008 | 2011 | 2014 | 2018 |
| --- | --- | --- | --- | --- | --- | --- | --- | --- |
| Prevalence (n, %) | | | | | | | | |
| Hypertension | 1,169(12.86) | 1,559(13.92) | 2,446(15.23） | 2,824(18.06) | 3,267(19.27) | 2,725(28.18) | 2,237(31.10) | 6,261(39.44) |
| Diabetes | 75(0.82) | 159(1.42) | 371(2.31) | 414(2.65) | 432(2.55) | 400(4.10) | 382(5.31) | 1,423(8.96) |
| Heart disease | 665(7.31) | 839(7.49) | 1,420(8.84) | 1,400(8.95) | 1,507(8.89) | 1,175(12.03) | 892(12.40) | 2,535(15.97) |
| Stroke or CVD | 292(3.21) | 439(3.92) | 851(5.30) | 849(5.43) | 1,015(5.99) | 802(8.21) | 606(8.43) | 1,654(10.42) |
| Chronic lung disease | 1,134(12.47) | 1,253(11.19) | 2,085(12.98) | 1,860(11.89) | 1,769(10.43) | 1,135(11.62) | 785(10.91) | 1,562(9.84) |
| Tuberculosis | 78(0.86) | 82(0.73) | 124(0.77) | 118(0.75) | 109(0.64) | 102(1.05) | 35(0.49) | 116(0.73) |
| Cataract | 1,694(18.63) | 1,441(12.87) | 1,922(11.97) | 1,895(12.12) | 1,872(11.04) | 1,156(11.84) | 839(11.67) | 2,048(12.9) |
| Glaucoma | 218(2.40) | 267(2.38) | 438(2.73) | 399(2.55) | 347(2.05) | 155(1.59) | 93(1.29) | 286(1.80) |
| Cancer | 48(0.53) | 34(0.30) | 65(0.40) | 70(0.45) | 75(0.44) | 83(0.85) | 61(0.85) | 205(1.29) |
| Prostate tumor | 332(3.65) | 282(2.52) | 344(2.14) | 516(3.30) | 593(3.50) | 437(4.48) | 324(4.51) | 677(4.27) |
| Gastric or duodenal ulcer | 293(3.22) | 376(3.36) | 813(5.06) | 791(5.06) | 849(5.01) | 381(3.92) | 258(3.59) | 671(4.23) |
| Parkinson’s disease | 86(0.95) | 53(0.47) | 73(0.45) | 77(0.49) | 86(0.51) | 75(0.77) | 47(0.65) | 123(0.77) |
| Bedsore | 78(0.86) | 82(0.73) | 148(0.92) | 126(0.81) | 93(0.55) | 55(0.56) | 38(0.53) | 70(0.44) |
| RR of multimorbidity | | | | | | | | |
| Hypertension | 5.60(5.07,6.18) | 5.45(5.01,5.92) | 6.58(6.15,7.05) | 5.94(5.59,6.32) | 5.06(4.79,5.35) | 4.14(3.91,4.39) | 3.86(3.63,4.12) | 3.25(3.14,3.38) |
| Diabetes | 32.67(17.43,62.34) | 23.01(15.88,33.36) | 12.86(10.31,16.05) | 17.44(13.84,21.96) | 15.34(12.37,19.03) | 16.50(12.78,21.29) | 17.11(13.08,22.37) | 14.91(12.89,17.25) |
| Heart disease | 15.99(13.58,18.82) | 13.25(11.60,15.14) | 12.63(11.35,14.06) | 14.60(13.02,16.37) | 13.48(12.12,15.00) | 12.10(10.63,13.77) | 12.59(10.81,14.68) | 10.14(9.25,11.13) |
| Stroke or CVD | 8.98(7.14,11.28) | 10.58(8.79,12.73) | 9.07(7.94,10.37) | 10.76(9.35,12.37) | 10.74(9.47,12.18) | 9.04(7.80,10.48) | 9.38(7.88,11.16) | 10.51(9.33,11.83) |
| Chronic lung disease | 5.45(4.93,6.03) | 5.55(5.03,6.11) | 4.72(4.37,5.08) | 5.39(4.97,5.84) | 5.09(4.68,5.53) | 5.47(4.90,6.11) | 5.49(4.80,6.28) | 4.76(4.31,5.26) |
| Tuberculosis | 13.55(8.36,21.97) | 11.26(7.21,17.59) | 14.79(9.92,22.07) | 16.90(10.95,26.06) | 12.55(8.30,18.99) | 15.02(9.15,24.67) | 13.44(7.78,16.66) | 13.20(7.99,21.79) |
| Cataract | 5.11(4.73,5.51) | 6.15(5.64,6.72) | 6.53(6.03,7.07) | 6.26(5.77,6.79) | 6.29(5.80,6.83) | 6.12(5.48,6.83) | 6.78(5.93,7.75) | 7.37(6.70,8.10) |
| Glaucoma | 8.21(6.31,10.69) | 9.56(7.54,12.14) | 10.29(8.47,12.50) | 10.35(8.41,12.72) | 11.04(8.83,131.81) | 11.37(7.85,16.48) | 7.17(4.64,11.09) | 15.35(10.97,21.48) |
| Cancer | 11.33(6.24,20.60) | 7.31(3.73,14.30) | 7.72(4.71,12.65) | 7.60(4.71,12.28) | 7.08(4.48,11.19) | 7.98(5.00,12.72) | 10.91(6.02,19.77) | 9.70(6.90,13.63) |
| Prostate tumor | 18.15(14.19,23.21) | 13.20(10.36,16.82) | 10.80(8.65,13.49) | 9.72(8.13,11.62) | 12.52(10.52,14.89) | 9.74(7.91,12.00) | 9.29(7.29,11.84) | 10.40(8.60,12.56) |
| Gastric or duodenal ulcer | 8.52(6.80,10.69) | 8.96(7.36,10.92) | 5.78(5.07,6.59) | 5.86(5.13,6.70) | 6.40(5.62,7.28) | 7.30(5.92,9.01) | 5.67(4.43,7.26) | 7.37(6.21,8.76) |
| Parkinson’s disease | 14.64(9.17,23.37) | 12.63(7.17,22.24) | 16.95(9.87,29.12) | 5.44(3.48,8.49) | 11.52(7.28,18.21) | 6.48(4.04,10.40) | 6.30(3.45,11.49) | 9.04(5.87,13.92) |
| Bedsore | 16.43(9.92,27.23) | 12.53(7.96,19.71) | 9.22(6.61,12.87) | 7.50(5.26,10.71) | 7.55(4.99,11.41) | 8.38(4.69,14.97) | 5.57(2.89,10.75) | 8.18(4.69,14.28) |

Prevalence of disease and RR of multimorbidity were presented as count (percentage) and percentage (95% CI).
